# 3D Printed Modular Immunofiltration Columns for Frequency Mixing-Based Multiplex Magnetic Immunodetection

**DOI:** 10.3390/s19010148

**Published:** 2019-01-03

**Authors:** Stefan Achtsnicht, Julia Tödter, Julia Niehues, Matthias Telöken, Andreas Offenhäusser, Hans-Joachim Krause, Florian Schröper

**Affiliations:** 1 Institute of Complex Systems Bioelectronics (ICS-8), Forschungszentrum Jülich, 52425 Jülich, Germany; s.achtsnicht@fz-juelich.de (S.A.); m.teloeken@fz-juelich.de (M.T.); a.offenhaeusser@fz-juelich.de (A.O.); 2 Fraunhofer Institute for Molecular Biology and Applied Ecology IME, 52074 Aachen, Germany; Julia.Toedter@ime.fraunhofer.de (J.T.); Julia.Niehues@ime.fraunhofer.de (J.N.)

**Keywords:** frequency mixing magnetic detection, 3D print, magnetic sandwich immunoasssay, additive manufacturing, magnetic bead

## Abstract

For performing point-of-care molecular diagnostics, magnetic immunoassays constitute a promising alternative to established enzyme-linked immunosorbent assays (ELISA) because they are fast, robust and sensitive. Simultaneous detection of multiple biomolecular targets from one body fluid sample is desired. The aim of this work is to show that multiplex magnetic immunodetection based on magnetic frequency mixing by means of modular immunofiltration columns prepared for different targets is feasible. By calculations of the magnetic response signal, the required spacing between the modules was determined. Immunofiltration columns were manufactured by 3D printing and antibody immobilization was performed in a batch approach. It was shown experimentally that two different target molecules in a sample solution could be individually detected in a single assaying step with magnetic measurements of the corresponding immobilization filters. The arrangement order of the filters and of a negative control did not influence the results. Thus, a simple and reliable approach to multi-target magnetic immunodetection was demonstrated.

## 1. Introduction

Different biosensing technologies have been established to determine qualitatively or measure quantitatively the presence of a target substance inside a sample solution, for example mass spectroscopy, surface plasmon resonance measurements, and chromatographic methods [1]. An important group of technologies is based on immunological methods. They have in common the fact that they are relatively fast, cost effective, robust and do not need an extensive sample preparation [2]. In immunologic technologies, the capture part for biorecognition is very often an antibody, but also phages, nucleic acids (Aptamers) or molecular imprinted polymers come to use [2]. This captured part can bind to different target substances such as bacteria, cells, spores, viruses or toxins [3]. Additionally these methods have a label connected to it, for instance enzymes, fluorophores, radioisotopes, or magnetic beads [3,4].

The most widely used immunoassay approach is the enzyme-linked immunosorbent assay (ELISA) [5]. One type of ELISA is the sandwich-based assay, where target-specific antibodies are immobilized to a surface—typically the cavities of a 96-well plate. Then the corresponding target is captured, if present in the sample. Subsequently, secondary antibodies are applied typically functionalized with enzyme labels, catalyzing a reaction that leads to a color change, which might be difficult to detect when the analyte is colored or turbid. A disadvantage of alternative markers like fluorophores or radioisotopes is that they have a half-life time or undergo photo bleaching which leads to a signal change over time. In contrast, the properties of the magnetic beads do not change with time and, therefore, can be read out again later [6].

Magnetic beads (MB) are widely used in modern bioanalytical and biomedical applications [1,7,8] either as a label, a carrier, or both. A variety of different magnetic sensors have been applied, for example hall sensors [9], coils, spin-valve sensors [10], anisotropic magnetoresistance sensors (AMR) [11], giant magnetoresistance (GMR) sensors [12,13], giant magnetoimpedance (GMI) sensors [14] and superconducting quantum interference devices (SQUIDS) [15]. Magnetic measurement techniques include relaxometry [16], susceptometry [17], nuclear magnetic resonance (NMR) [18] or magnetic frequency mixing [19,20], the technique we use in this work.

Immunoassays can either be performed in a homogeneous format [21], e.g., magnetic relaxation immunoassay (MARIA) [22] or immunomagnetic reduction (IMR) [23], or by immobilization on a surface in a sandwich assay format [24,25]. The latter have the advantage that they can be very specific and sensitive, especially if done with magnetic labels [26]. In contrast to standard ELISA, the primary antibodies are not bound to a flat surface but inside the pores of a 3D-immunofiltration column, leading to a substantial increase of the reaction surface and thus to higher signals.

Usually, with such sandwich immunoassays, only one analyte can be detected. In many cases, like, for example, the diagnosis of certain diseases, it would be desirable to detect and quantify different biomarkers at the same time [4,27]. Several approaches have been undertaken to realize a so-called multiparametric immunoassay [28] by means of distinction of different types of magnetic beads or by binding in different detection regions [29]. Distinction of different types of beads has been shown experimentally [30,31], however a real magnetic immunoassay with simultaneous detection of several analytes still remains to be shown. Therefore, we chose the approach of a modular multi-filter assay with sequential magnetic readout of the different filter compartments. The filters are functionalized with different antibodies for selective binding of different analytes.

3D printing was used as a method of choice for building customized modular detection compartments because it offers some features that are good for our purpose. Particularly the quick iterative design, production and verification process can be mentioned as well as the production of small series components [32]. It has been shown that 3D printing can be used for different purposes in natural sciences [33,34] like printing of laboratory equipment and components (e.g., a 24-well plate), supporting devices, housings, microfluidic devices and optical components or (medical) phantoms [35] or on-site chemical manufacturing of fine chemicals and pharmaceuticals [36].

## 2. Materials and Methods

### 2.1. 3D Printed Filter Holders

All filter holder parts were designed with the computer aided design (CAD) software Autodesk^®^ Inventor 2018 and 2019 from Autodesk, Inc. (San Rafael, CA, USA) and later exported as ASCII STL-File with the resolution option set to high in millimeters.

We chose the stereolithography (SLA) printing technology because we wanted to have a very smooth surface at the inside, and a transparent structure to be able to verify the positioning of the filter optically and to observe the homogeneity of the flow and the enrichment of the filter with bound magnetic beads which have a brownish color. Additionally we wanted a possibility to manufacture the parts with repeatable precision and without complicated pre-treatment.

We used a Form 2 printer from Formlabs Inc (Somerville, MA, USA), operated with the most current firmware version (rc-1.18.12-58). The 145 × 145 mm [37] sized building base enabled us to print many of our designed parts in one printing run which is advantageous for testing of different samples and infield use.

All parts were printed using Formlabs’ Clear Resin RS-F2-GPCL-04 [38,39]. It consists of a mixture of methacrylic acid esters and photoinitiator [40] which turns from liquid to solid due to the laser light of the printer. According to its material datasheet [38], it shows a weight gain of less than 1% in 24 h for water, salt water or isopropyl alcohol, which are similar liquids to those our printed parts will be exposed to in their later use. The printer resin combination allows a resolution of 0.025 mm along the *z*-axis, which was sufficient for our experiments. As the print time scales inversely with the layer height, the prints took significantly longer with these finer layers, but led to a smoother cone structure, as explained in [41]. Prints with this resin can be almost transparent even without post-treatment and, therefore, enable the optical observation of what happens inside the parts.

The STL-Files of the constructed parts were imported into PreForm V2.17.0 software from Formlabs (Somerville, MA, USA). All parts were orientated upright because in our tests, we found that this orientation leads to a more round outcome and less supporting structure is needed. The parts were oriented such that the support structure was just connecting to the top of the female cones. Possible residues during cleaning did not affect the fitting inside the measurement head (which would be the case if the support structure contacted the side walls) or inside each other (which might happen if the supports were on the tips of the male parts). The support structure was at first auto-generated by the software with the option of no internal support structure, because they would be hard to remove and residues would affect the roundness of the inner parts. Later, the auto-generated support structures were modified so that no support structure is placed at points that are critical for the latter fitting of the parts into each other or that could disturb the liquid flow.

After printing, the parts were washed and ultraviolet (UV)-cured with Formlabs’ Wash and Cure System according to the recommendations by the manufacturer. All support structures were removed using flush cutters and a scalpel. Afterwards the parts were stored under ambient lab conditions until their final use in the experiment.

### 2.2. Magnetic Sandwich Immunoasssay

Magnetic beads (Article-No. 104-19-701, Lot. 04117104) with a 75 nm hydrodynamic diameter (Z-Average, Polydispersity index: 0.12) were purchased from micromod Partikeltechnologie GmbH (Rostock, Germany). They have a superparamagnetic core and a functionalized shell with streptavidin bound to the surface. They exhibit a biotin binding capacity of 434 pmol. All given characteristics are according to the manufacturer. These beads were chosen because they yield a strong signal and are small enough to easily pass through the immunofiltration matrices.

In this research we used recombinant monoclonal human antibody rAb M12 [42] and monoclonal murine antibody mAb 54k [43], both generated by Fraunhofer IME as analyte in a sandwich assay approach.

#### 2.2.1. Preparation of the Columns and Filters

To perform the sandwich immunofiltration assay, we used a modified protocol from [44]. We changed to a batch method for immobilization of the analyte-specific capturing antibodies. With this, it is easier and faster to prepare many matrices at the same time, in contrast to the flow-through method.

The 3D printed parts were placed inside a beaker with phosphate-buffered saline, pH 7.4 (PBS) containing bovine serum albumin (BSA) [10 mg/mL] to saturate their surface and prevent unspecific binding of the target substances or magnetic beads during the immunoassay preparation.

We used hydrophobic porous polyethylene (PE) matrices (hydrophobic ABICAP HP filter 5 × 5 mm) purchased from Senova Gesellschaft für Biowissenschaft und Technik mbH (Senova, Weimar, Germany), which are also used in their ABICAP detection system. The matrices are cylinders with a diameter and height of 5 mm, respectively, and a mean pore size of 20 µm.

Since we need to place the filters inside our 3D printed filter holders anyway, and they are not prepacked like the commercially available ABICAP columns, we decided to switch from the previously used gravity-driven flow-through antibody immobilization procedure to a batch method. As a first step, the matrices were hydrophobically equilibrated to remove all air from the pores and to later achieve a homogeneous antibody loading. Therefore, the matrices were placed in a beaker with ethanol (96% vol/vol) inside a desiccator for 1 h with an underpressure of −0.8 bar. Then they were washed inside a tube in consecutive steps with an ethanol–water mixture (50/50), MilliQ water and then 0.1 mM carbonate buffer as immobilization buffer [100 mM NaHCO_3_, 33.6 mM Na_2_CO_3_, pH 9.6], each time for one hour with the help of an overhead shaker. It was ensured that the tubes are filled with liquid up to the top and closed without trapped air.

The filters were then placed inside a small tube with 0.1 M carbonate buffer with 10 µg of the corresponding polyclonal catching/primary antibody (GAH-FC or GAM-FC, Jackson ImmunoResearch Europe Ltd., Cambridgeshire, UK) per matrix, or for negative control without antibody, using 10 mg/mL biotin free BSA (Albumin Fraktion V, Carl Roth GmbH + Co. KG, Karlsruhe, Germany), and incubated overnight at 4 °C with an overhead shaker. Again it was made sure that no air was remaining inside these tubes. During overnight incubation, the antibodies can bind to the matrix by diffusion through the pores and by self-organized hydrophobic interaction. Afterwards the matrices were washed with PBS to remove unbound antibodies.

The filters were pressed into the 3D printed matrix holder with homogeneous pressure using a push rod which was printed a few µm smaller in diameter than the matrix. To saturate uncoated parts of the matrices and holders and, therefore, prevent unspecific binding of substances, the columns were incubated with BSA in gravity flow. Hence, all holders were filled to the top with a BSA solution [10 mg/mL in PBS] and after the gravity-driven flow they were incubated for 20 min at room temperature. This step was repeated two times, so that they had a total of three saturation runs. Subsequently, the matrices were washed with PBS and the modular immunofiltration sample holders were plugged together in stacks of three in the desired combination. To achieve an even flow, the holders were filled with PBS before plugging them together, and flushed in gravity flow with PBS. On the bottom, an also-printed Luer connector was added, the system was flushed and filled with PBS, and then closed with a plug on the bottom to keep the whole setup filled and the matrices wet. The columns were stored at 4 °C until use in the experiment.

#### 2.2.2. Performing the Magnetic Immunoassay

For performing the immunoassay experiment, the bottom parts of the columns were opened. During the assay, all liquids were flushed through the multimatrix columns by gravity flow.

First, 500 µL of the prepared analyte solution (500 µL PBS + 1 µg rAb M12 + 1 µg mAb 54k, 500 µL PBS + 1 µg rAb M12 or 500 µL PBS + 1 µg mAb 54k) was added, followed by application of two times 500 µL PBS to make sure that the analyte solution is flushed through all three matrices. Afterwards it was incubated for 15 min, followed by two washing steps with 500 µL of PBS each, to remove unbound material.

In the next step, a mixture of biotinylated secondary antibodies with binding specificity to human antibodies (5 µg GAH-λ-biotin (Rockland Immunochemicals Inc., Limerick, PA, USA)) and murine antibodies (5 µg GAM-F(ab)_2_-biotin (Jackson ImmunoResearch Europe Ltd., Cambridgeshire, UK) solved in 500 µL PBS) was added and flushed through the columns, followed by application of two times 500 µL PBS. Samples were incubated for 15 min, before three washing steps with 500 µL PBS each were performed.

**Figure 1:**
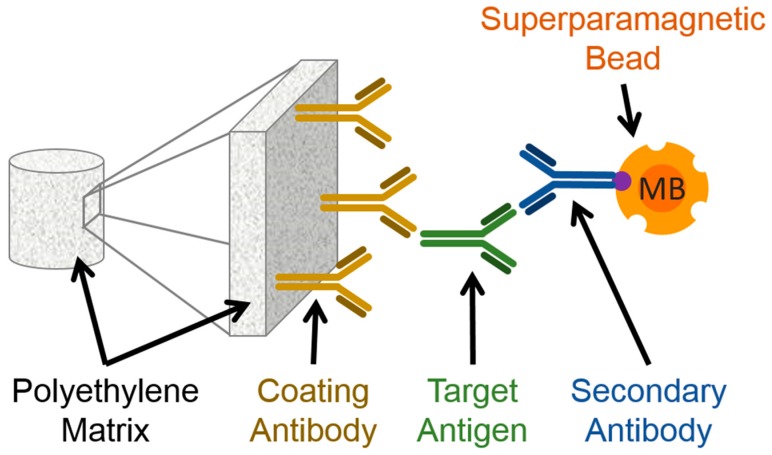
Schematic representation of the magnetic sandwich immunofiltration assay. A coating antibody (also primary antibody, here GAH-Fc or GAM-Fc) is immobilized on a polyethylene matrix. The target antigen (rAb M12 or respectively mAb 54k) is captured by the antibody when the antigen-containing solution is flushed over in gravity flow. As the next step, the secondary antibody (GAH-λ-biotin or GAM-F(ab)_2_-biotin respectively) is flushed over, which also binds to the target antigen. Finally the streptavidin-coated superparamagnetic beads (MB) bind to the biotin moiety of the secondary antibodies.

As a last step before the measurement, the magnetic bead (MB) solution (180 µg MBs + 500 µL PBS) was flushed over the columns in gravity flow, forced through all matrices by applying two times 500 µL PBS, incubated for 5 min, and washed three times with 500 µL PBS-T (0.05% Tween20) to remove unbound MBs. This is important because remaining unbound MBs would lead to a false positive measurement signal. A schematic representation of this sandwich immunofiltration assay is shown in Figure 1.

Finally, the columns were moved through the measurement head of the reader, each matrix was read out, and the values obtained in mV were recorded.

### 2.3. Magnetic Frequency Mixing Detection

**Figure 2:**
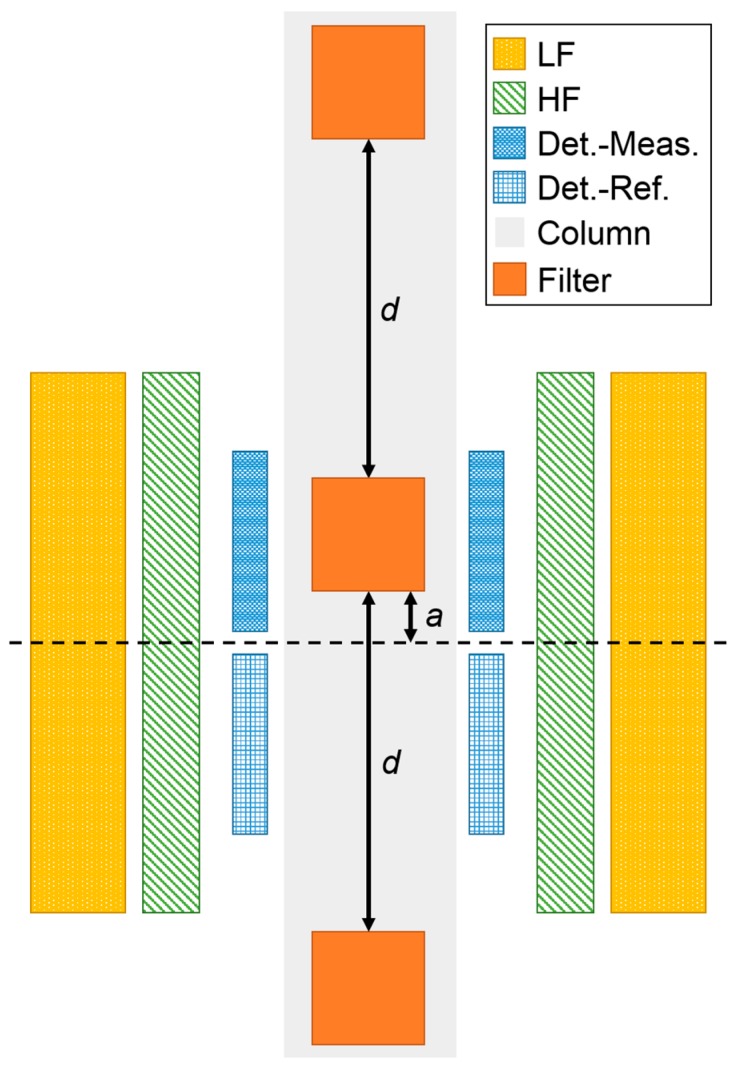
Schematic drawing of an axial cut through the multi-filter setup inside the measurement head. The measurement head consists of two coils for providing the oscillating low (LF yellow, dotted) and high (HF, green, diagonal lines) frequency magnetic field. The differential detection coil is shown with its two compartments for measurement (fine blue grid, top) and reference (blue grid, bottom). Three filters (orange) inside a column are shown. They have a distance *d* between each other. For the filter in the middle, the optimal distance *a* is indicated as well.

In this work, we use the magnetic frequency mixing technique to detect the magnetic markers, as described in [20]. Therefore, we use the same magnetic handheld reader device as described in [44]. The measurement head consists of three coils. One is used to apply the sinusoidal magnetic driving field with a frequency *f*_2_ of about 61 Hz. This driving field with a maximum amplitude in the range of a few mT is strong enough to saturate the superparamagnetic beads at the extrema of the driving field. The second coil generates a magnetic excitation field (about 49 kHz) which is used as a probing field for the magnetization state of the beads. It generates a magnetization and, therefore, a strong response when the driving field is close to zero, whereas at the extremes of the driving field where the particles are already saturated, the response to the excitation field is weak. Thus, the response amplitude to the excitation field oscillates with a frequency 2·*f*_2_. Since the employed magnetic beads are superparamagnetic [8], meaning that they exhibit a non-linear, non-hysteretic behavior if they are exposed to a magnetic field, new frequencies are generated, following the scheme: *f*_new_ = *m*·*f*_1_ ± *n*·*f*_2_, where *m* and *n* are integer numbers. The third coil is used as detection coil to pick up the induced signal. It is a differential coil consisting of two coils with identical parameters (e.g., number of windings, diameter) but opposite winding directions. The two parts of the detection coil are called the measurement and reference compartments. The detection coil is placed in the center of the two excitation coils of the measurement head (compare Figure 2). During a measurement, the sample containing superparamagnetic beads is positioned inside the measurement compartment, the reference compartment is empty. By using this differential coil, the very strong direct induced signal is cancelled out, whereas the sample’s signal is just induced in one coil and, therefore, does not cancel out. This signal is then amplified, filtered and the amplitude of the response component *f*_1_ + 2·*f*_2_ is demodulated in a two-stage multiplication process. This frequency-mixing component is chosen because it exhibits the strongest mixing signal without the presence of a static magnetic offset field. A single measurement takes about 15 s in total. This includes 12 s waiting time in the beginning for reaching equilibrium, followed by ten measurements within 3 s. The mean value and standard deviation of the signal amplitude is then displayed on a touch screen or transmitted via USB to a connected PC.

## 3. Results

### 3.1. Design Approach

Our measurement head (Figure 2) is specifically designed to be used with ABICAP columns, enabling the detection of magnetic particles specifically enriched within its immunofiltration matrices.

We decided to place the filters at such a distance that the recorded signal is only affected by the beads inside the currently measured filter of interest, and not by those from adjacent filters. With this approach, very small and big signals in adjacent matrices can be distinguished and liquid handling is kept as simple as possible. The filters can be placed in the most sensitive area of the coil, as designed for ABICAP columns, and the results can be directly compared.

### 3.2. Simulation and Design

As mentioned before, our detection coil (Figure 2) consists of two serially connected coils that are oppositely wound to cancel out directly induced signals. We need to ensure that while one filter is measured, none of the others is within the detection range of either the measurement coil or the reference coil.

To determine the required minimum distance between two matrices, we wrote a program in Python 3 which allows to calculate the sensitivity of each position inside a given measurement head and determine the signal that an axially symmetric sample would produce at different positions inside our measurement head. This sensitivity at each point is a result of the sensitivity of the measurement part of detection coil minus the sensitivity of the reference part of the detection coil, weighted with the signal amplitude generated by the excitation coil and the sample’s concentration. The software works in a way that the sample is sliced into different layers, and each layer is spliced into individual points. For each point, the signal is calculated, weighted with the corresponding sensitivity, and the integral over all points are computed. The distances between the layers and points can be set to the desired accuracy of the calculation. With a smaller distance between these grid points, the calculation becomes more precise but the calculation time gets longer. The position of the sample along the *z*-axis is varied until the maximum of the signal is found.

**Figure 3:**
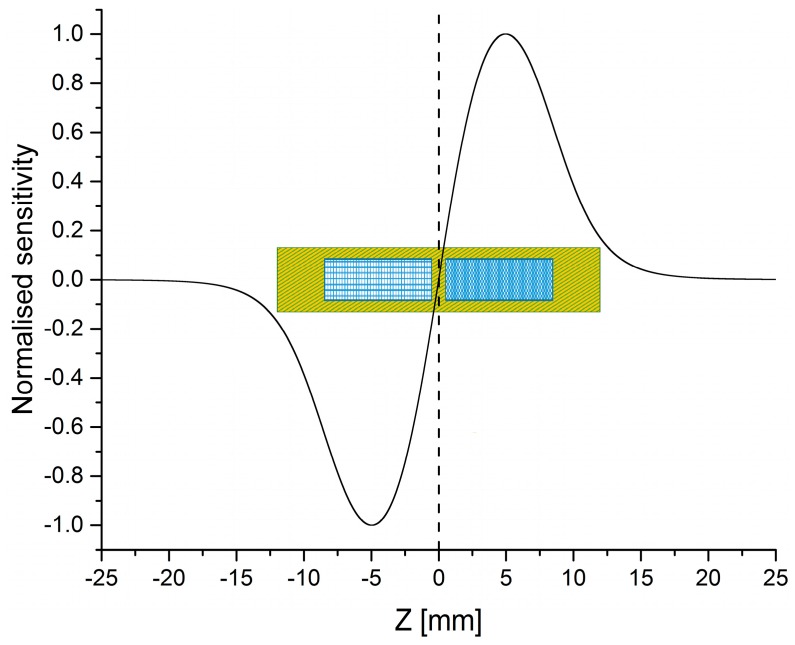
Simulated normalized sensitivity profile of the measurement head shown in Figure 2. Additionally the Z-positions of the differential detection coil with its two compartments for measurement (fine blue grid, right) and reference (blue grid, left) as well as the excitation coils (HF + LF, green-yellow, diagonal lines) are indicated.

The function of this software was verified with previous manually calculated results and by comparing the calculated sensitivity profile (see Figure 3) with that obtained from scanning a mm-sized sample of highly concentrated MBs sample across the detection coil. The software is not limited to these filter matrices. For example, it has also been used to calculate the amount of liquid inside a given pipette tip geometry which is placed inside the measurement head at which the ratio of signal to required volume is maximal. By applying this software in the reverse way, it is possible to compare the signal of different samples even when they are measured in different sample holders.

**Figure 4:**
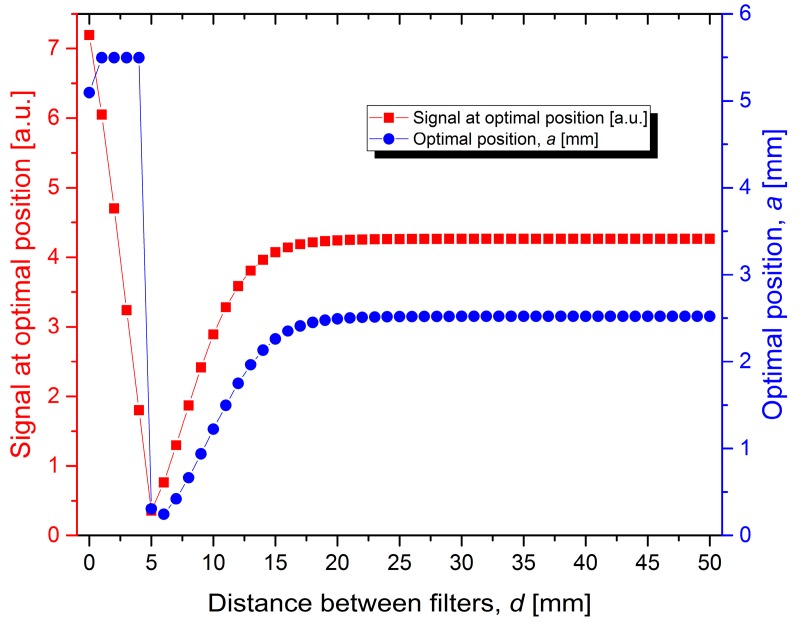
Simulation result for 3 filters (5 × 5 mm) with a grid point-to-point distance of 0.01 mm in the measurement head. Shown is the optimal position *a* (blue circles) and the value at this optimal position (red squares) as a function of the spacing *d* between the filters. This distance d is swept in steps of 1 mm from 0 to 50 mm.

The determination of the minimum filter spacing was performed with a script written for this purpose. We simulated an arrangement of 3 filters (cylinders with 5 × 5 mm and a uniform loading) whose distance *d* between them could be set. In Figure 2, the excitation and detection coils and the three filters are illustrated. The point-to-point distance inside the sample was set to 0.01 mm. The distance *d* between adjacent filters was swept from 0 to 50 mm with a step size of 1 mm. For each distance *d*, an optimization was performed to find the position inside the measurement coil at which the filter in the middle gives the highest signal (which will now be called the optimal position *a*). At least three filters had to be used because it needs to be ensured that the distance between the filters is chosen such that the filter on top does not affect the signal in the measurement part of the detection coil, while the filter below should not affect the signal in the measurement and the reference part of the detection coil. To find the minimal distance between two filters, the signal at the optimal position and the optimal position itself were plotted as a function of the distance between the filters. The results are shown in Figure 4. It can be seen that the signal at the optimal position (red squares) is at the beginning higher than at longer distances. This is the fact because when there is no or just a very short distance between them, also the outer filters are contributing to the total signal (compare to Figure 2). When the distance is increased, the signal becomes smaller. This happens when the filter in the middle is inside the measurement coil, the signal of the filter below will be detected by the reference coil and is therefore reducing the total measured signal of this differential detection coil. At longer distances, the signal starts to increase again because the lower filter is now getting further away from the reference coil so that its negative signal becomes weaker. For longer distances, it is approaching a fixed value. By observing the optimal position (blue circles) for each step, it can be seen that at the beginning, the optimal position is such that the filters are as high as possible and the lowest filter produces the smallest possible signal in the reference coil (see also Figure 2). When this is not possible any more, the position is varying and later getting steady again when both other filters do not affect the total measured signal.

The 3D printed parts were designed so that they can be stacked on top of each other and the calculated minimum distance is respected. Therefore, we added a male cone in the center of the lower part of the filter holder which has an outer angle of 3° equal to the one of a Luer adapter but with a bigger diameter. There is no step between the inner diameter of the filter (5 mm) to the 2 mm outlet diameter because we designed a small cone here which guides the liquid. For the top of the stackable matrix holders, a female Luer adapter was designed. As we need to be able to add the filters inside our sample holder, we could not directly work with Luer connections because they are too small for the 5 mm filters. To hold the filters apart, two solutions are practical: one is that the distance part has just a thin inner diameter and so less liquid is needed to fill it completely without air left inside. If some air gets into the piping system, e.g., due to media exchange, it cannot collect over the filter and, therefore, has to go through it. The other possibility is to make the part between the filters as wide in diameter as the filter. This design needs more liquid to be completely filled, but if some air gets into the system, it can collect in these regions and does not need to go across the filter. As air inside the filter leads to varying pressures and blocked parts which then leads to different flow rates (=different incubation times) or uneven and unreproducible binding inside the filter, we decided to choose the way where the distance parts between the filters are as wide in diameter as the filter for a long distance. To still be able to use the filter parts inside the standard Luer system, additionally we created two adapters: one from the female connector of our system to the male Luer cone, and one from female Luer cone to the male plug of our system. This one also has a small cone at the beginning for easier centering the male Luer adapter before entering the female part which makes the connection much easier, especially if used together with an automated system.

### 3.3. 3D-Printing and Verification of the Design and Print

After the optimal filter distance was identified in simulation, we started the CAD process and printed the stackable column parts as described in Materials and Methods.

The inner diameter of the filter holder is the most crucial size of the setup. Therefore we used the dimensions of the filter given by the manufacturer and compared them with measurements with a sliding caliper. This was a good starting point for the construction, but needed to be adjusted corresponding to the result after printing. For example, the shrinking rate between different materials and printing methods might be different. To speed up this process, we constructed and printed gauges which had different inner diameters to find the size of interest. After printing a filter holder, it was verified that the filter fits tightly inside. We verified that the fluid runs smoothly through the filter and does not pass between filter and the wall. This procedure was repeated until the results were comparable to the commercially available ABICAP columns. All filters used for these experiments were hydrophobically equilibrated to have them in the same configuration as used for the immunoassay. This was done to prevent changes of the filter, for example enlargement due to the wetting, spoiling our test results.

**Figure 5:**
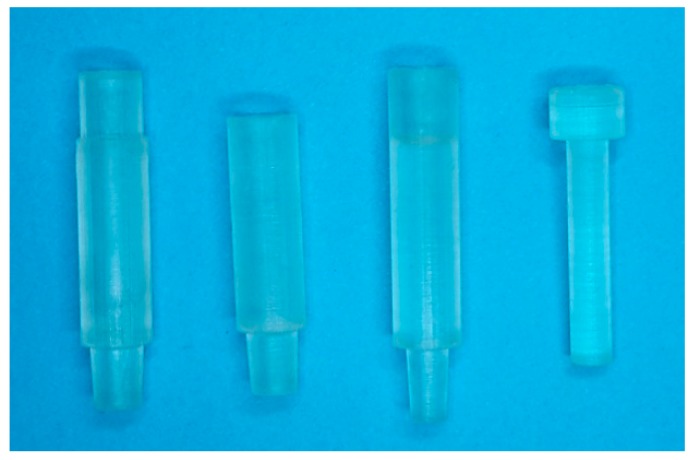
Picture of the 3D printed parts used in this research. Shown are from left to right: female Luer to filter holder adapter, filter holder, filter holder to male Luer adapter and the push rod for placing the filters inside the filter holders. The outer diameters of the filter holder as well as the adapters are 7.5 mm.

The Luer adapters were tested to ensure they fit to syringes and other commercially available Luer connectors (male and female). To ensure that filters were not damaged during insertion inside the holder when applying high pressure at one point, we printed a push rod which is just a few hundred micrometers smaller than the inner diameter of the filter holder. The printed parts are shown in Figure 5.

### 3.4. Results of the Multiplex Magnetic Immunoassays

**Figure 6:**
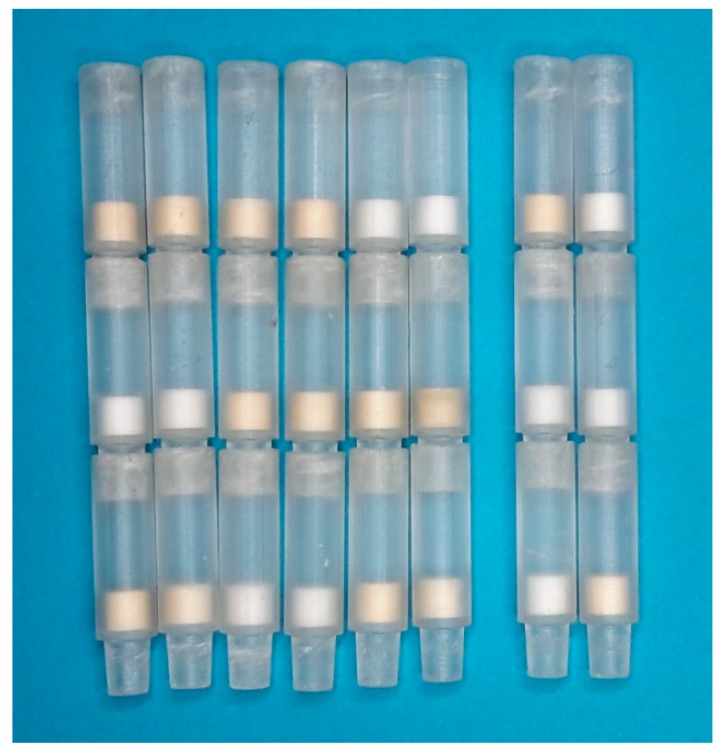
Picture of the stacked multi-filter columns. They are ordered in the same way as their measurement signals are shown in Figure 7. The left six columns (Column 1–6 in Figure 7) contain all possible combinations of 3 different coated filters, each flushed with both target antigens. The two columns on the right (A and B in Figure 7) are the ones where just one analyte was flushed over, they are stacked in the same order as column 1. All the filters are 5 mm high, and the outer diameters of the filter holder are 7.5 mm.

When performing the magnetic immunofiltration assays, it could be seen that there is a good seal between the individual stackable columns and that the MBs are washed over the matrices with a flow profile which is comparable to that observed when just one matrix is used in the original ABICAP column. As the whole 3D printed column remains transparent and did not get a brownish color, it could also be verified that the beads did not bind directly to their BSA blocked surface and that the brownish color is spread homogeneously over the whole filter. Figure 6 shows a photo of all columns after performing the assays.

**Figure 7:**
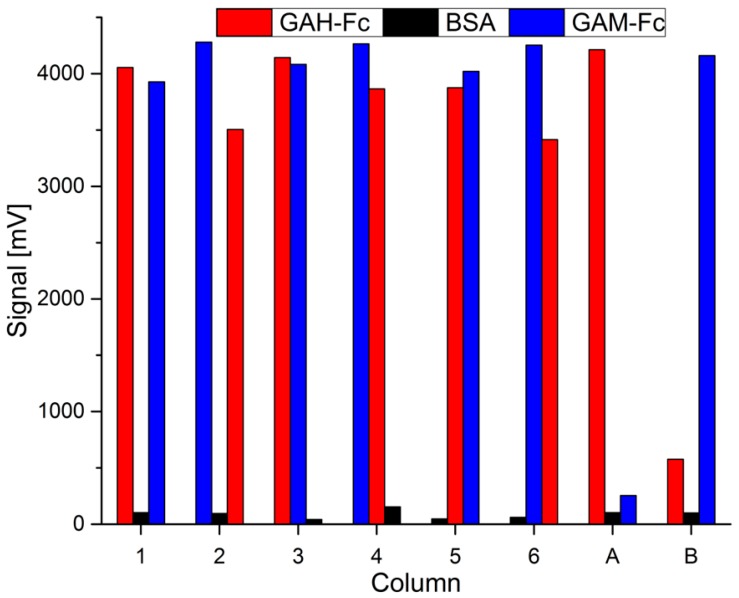
Graphical representation of the measurement signals. Standard deviations are too small to be depicted. All six possible combinations of three different filters were prepared as individual columns and used in an assay. They are marked here as 1–6. The measurements where only rAb M12 or mAb 54k was used as single analyte are marked as A or B, respectively. The order of the filters inside one measurement run is expressed in the order of the columns from left=top to right=bottom, while the immobilized capture antibody is shown by the color of the column.

To verify that the antigens bind specifically to one of the coating antibodies and to get an impression about the signal strength when there is no other antigen which could lead to cross reaction, we used two identical stacked columns with three different filters, but flushed them with a solution that just contains one of the antigens. They are marked as A (only rAb M12) and B (only mAb 54k) in Figure 7. Additionally the assay was performed with an analyte solution containing both target antigens with all six possible combinations of three different coated filters. The measured values are displayed in Figure 7, marked with the numbers 1–6. The type of capture antigen is color-coded, while the order of stacking from top to bottom is expressed as their order from left to right. A picture of all eight columns, in the same order as in Figure 7, is shown in Figure 6. The brownish color of some filters is due to enrichment of brownish colored magnetic beads.

**Figure 8:**
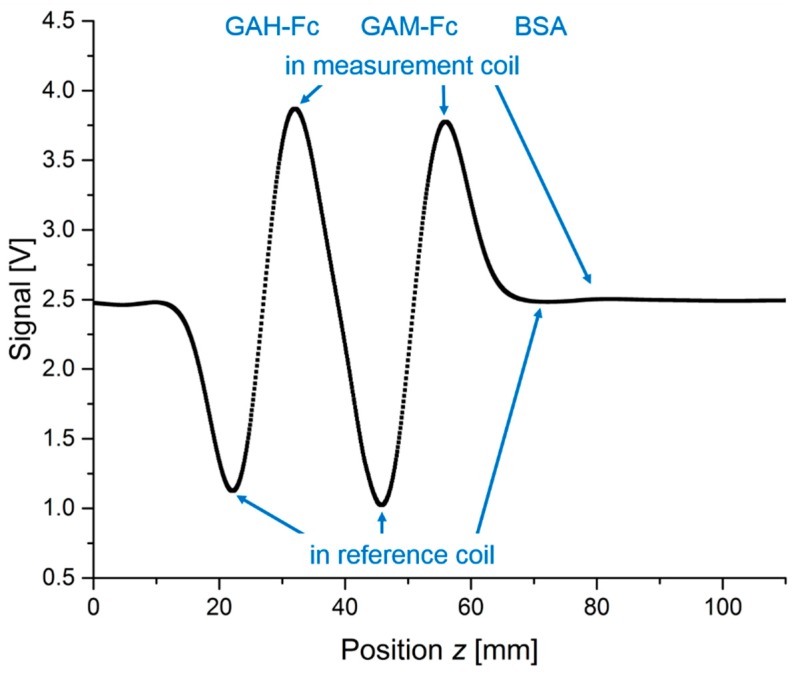
Measurement signal while column 3 is moved through the measurement head. The mean value for each point is shown, the standard deviation is smaller than the size of the data points. Every 0.1 mm, a measurement was performed. The offset of the reader was set so that the signal of the sample can be recorded no matter if the sample is inside the measurement or the reference coil. When the signal is lower than the offset, it means that the sample is inside the reference coil, while higher signals mean that more signal-delivering material is inside the measurement than in the reference coil.

All columns were moved through the measurement head in steps of 0.1 mm by means of a motorized vertical stage. At each step, the magnetic measurement was performed and the signal value was acquired. Figure 8 shows the scan of column 3. We selected this scan because two filters exhibiting strong signals are close together which allows a good characterization of the signals as well as identification of potential interfering effects during our scan. For this measurement, the reader was modified so that it has a constant offset in the middle (2.5 V) of the detection range (from 0 to 5 V), and the excitation amplitudes were reduced so that the signal never exceeds this range. An offset in the middle of the detection range is needed because during this scan, the MBs can be either inside the measurement or inside the reference part of the detection coil, delivering positive or negative signals, respectively. A signal smaller than the offset is related to the fact that more MBs are inside the reference than the measurement part of the detection coil.

## 4. Discussion and Conclusions

As expected, the simulation showed that the distance between the filters plays a crucial role in the resulting signal strength (compare Figure 4). It is important to place the filters at a distance where both parts of the detection coil do not pick up significant signals of the MBs in neighboring filters. It could also be seen that the required distance is bigger than the sum of the distance between both coil parts and the length of the reference coil. This can be explained by the fact that the oscillating magnetic field of the field excitation as well as the sensitivity of the detection coil does not become zero directly at the end of the coil, as can be seen from the simulated sensitivity profile in Figure 3. The scan of column 3 (Figure 8) shows that neighboring filters do not significantly influence the results because the signals in measurement and reference coil are almost identical in height, thus confirming the calculated distance between the filters. If there was a neighboring effect, we would see a reduction of the positive GAH-Fc peak and the negative GAM-Fc peak because of their partial overlap. The fact that we observe almost identical positive and negative peak heights for each antigen is an experimental confirmation of our calculated minimum filter distance.

3D printing with an SLA printer was found to be a suitable method for the construction of the stackable multi-filter columns presented in this work. The inner diameter of the stackable filter holder could be adjusted such that a tight fitting of the filter was realized, with the result that a homogeneous flow of the applied solutions through the filter was achieved. The designed cones led to a tight fitting between filter holders as well as the Luer connectors. From the simulation, the minimum distance between filters was obtained which yields clearly distinguishable peaks of the different filters in the measurement. The 3D printed parts were able to withstand the conditions during the preparation of the measurement (e.g., filling with BSA solution, flow-through of the antigen-/antibody- as well as the MB solution) and the subsequent read out of the columns. For example, the temperature inside the measurement head during readout is higher (around 40 °C) than room temperature, due to resistive heating of both excitation coils.

By observing the optical impression of the columns (Figure 6) as well as the signal trace in Figure 7, it can be seen that the MBs are concentrated inside the filters with the corresponding antibody, and not on the sidewalls or at the connections of the filter holders. This observation means that the blocked surface is not attractive to the MBs and target antigens. Therefore it can be pointed out that BSA blocking of this 3D printing material is an effective method to prevent surface binding of target and secondary antigens as well as of MBs.

The BSA blocked filters show no color change. In contrast, the filters where target antigens and MBs were able to bind, do change color. This qualitative optical impression was verified by comparing the measured values (see Figure 7) in the filters or the signal trace in Figure 8. Because of this, it can be followed that the blocking of the filters with BSA worked well.

As all filters were flushed with BSA before the analyte solution was applied, and the MBs can only bind in those filters where both the target antigen and the secondary antibody were previously enriched, it can be concluded that the batch method for coating has worked and the sandwich based immuno-binding was achieved successfully. This batch coating method enables a much easier and faster production of the columns in comparison to the gravity flow method. For us, it was particularly useful because we had to place the filters inside the printed filter holders anyway.

By comparing the signal strength of the two immunoassays where just one antigen was applied (Columns A and B in Figure 7) with the tests where both antigens (marked as 1–6 in Figure 7) were flushed through, it was experimentally confirmed that comparable signals could be observed.

From the results of columns A and B (compare Figure 7), it can be assumed that there is a slight cross reactivity between both used antigens/secondary antibodies. Matrices coated with the capture antibody which is not specific for the respective applied antigen showed slightly higher signals than those obtained from the completely BSA-saturated filters. Such a cross reactivity could be due to unspecific binding between the used antibodies, e.g., GAH might bind weakly to mouse antibodies, or GAM to human antibodies. Additionally it is possible that an unspecific protein–protein interaction occurs, for example between the immobilized coating antibodies and the streptavidin-coated MBs. However, the cross reactivity is small because the signals of the filters with the improper antibody are still much smaller than the signal in the filter with the correct target antibody. The signal of the filter with the specific bound antigen is 17- (column A) or seven-fold (column B) higher than the one of the unspecific binding. In the measurement where both antibodies are present, a similar cross reactivity cannot be excluded as well but does not contribute significantly to the high overall signals obtained from specific immunobinding. Thus the measured signal values fit very well to those of the reference measurements A and B.

By comparing the measured values of the filters with bound antigens to the BSA-blocked filters, or with those where the corresponding antigen was not contained, it can be seen that the filters with a bound antigen showed quite comparable values. It needs to be mentioned that the mean GAH-Fc signal is 11% lower if the GAH-Fc filter (red column in Figure 7) is located below the GAM-Fc filter (blue column). Similarly, the mean GAM-Fc signal is 6% lower when placing that filter below the other. Even though the MBs are applied in excess, there might be a slight reduction effect due to MB depletion or unspecific binding of the used antibodies or beads.

The coating and secondary antibodies we used are specific to human or murine antibodies. Hence, similar results should be achievable by using other antibody and antigen combinations of this kind. By changing the coating and secondary antibody, the same setup could be used to detect different targets inside a liquid sample. If more different targets need to be detected, i.e., more modular filtration compartments can be stacked together and the amount of liquids during the assay needs to be adjusted so that all constituents are flushed over all filters in each step.

In this research, we tested all possible combinations with this setup as well as the treatment with just one of each analyte as a comparison. In future work we intend to select a fixed order of matrices and record complete calibration curves for different analytes at various concentrations.
